# Student Use of Digital Patient Cases May Improve Performance in a Pharmacy Cardiovascular Therapeutics Course

**DOI:** 10.3390/pharmacy13020031

**Published:** 2025-02-21

**Authors:** Paul J. Wong, Noam Morningstar-Kywi, Rory E. Kim, Tien M. H. Ng

**Affiliations:** Alfred E. Mann School of Pharmacy and Pharmaceutical Sciences, University of Southern California, 1985 Zonal Ave, Los Angeles, CA 90089, USA; nmorning@usc.edu (N.M.-K.); rocallag@usc.edu (R.E.K.); tienng@usc.edu (T.M.H.N.)

**Keywords:** digital patient case, educational technology, instructional tool, pharmacy education, student performance, virtual patient case

## Abstract

The use of digital patient cases (eCases) is associated with student-perceived improvements in learning. However, novel instructional tools must demonstrate measurable student benefits to justify ongoing use. This research sought to identify the impact of digital patient cases (eCases) on student performance in a PharmD cardiovascular course. Optional eCases for hypertension (HTN), venous thromboembolism (VTE), and acute heart failure (AHF) were incorporated into the course. Performance on the exams and course overall was compared between student cohorts based on eCase use. Aggregated data were analyzed by year. Additional analysis was performed for scores on exam items related to eCase content. From 2020 to 2022, a total of 322/562 students (57.3%) used any eCase. While there were no differences in 2020 and 2021, eCase users in 2022 had significantly higher course (83.6% vs. 79.7%, *p* = 0.002) and final exam scores (75.0% vs. 67.7%, *p* < 0.001) compared with non-users. VTE eCase users had higher scores on VTE exam items compared to non-users, but only in 2021. AHF eCase users received higher scores on AHF exam items compared to non-users in 2021 and 2022. Among certain cohorts, student eCase use was associated with improved performance, and the use of certain eCases showed differences in content-specific performance. The eCase is a promising instructional tool that warrants further investigation to determine best design elements for maximal effectiveness.

## 1. Introduction

Students in healthcare professions are expected to graduate from their programs prepared to engage in clinical decision-making using sound clinical reasoning and critical thinking skills. Teaching these skills to medical and pharmacy students can be challenging as they sit at the highest level of Bloom’s Taxonomy [1,2,3]. Although health profession programs typically relegate the teaching of clinical decision-making skills to the experiential setting, this may not be the optimal approach [3]. Pharmacy students often require these skills to succeed in advanced pharmacy practice experiences and therefore need to develop them before rotations. In the pre-experiential setting, educators in medical and pharmacy schools use case-based learning to engage and guide learners through the clinical thought process and create a framework for approaching future problems [3,4,5,6,7]. However, case-based learning appears to have the most success with small groups, and instructors may find it difficult to design and facilitate these activities in large classrooms [5,8]. Furthermore, in the large classroom setting, it may not be possible for each student to receive individualized feedback from the instructor. Students may also look to reinforce their learning with further practice cases outside the classroom but lack the tools to do so. These challenges can hinder the success of case-based learning in teaching students valuable clinical reasoning and critical thinking skills.

The use of interactive, digital patient cases (eCases) that students can access on their electronic devices addresses this problem by allowing learners to individually practice their clinical decision-making skills in a safe-to-fail environment [9]. The design and implementation of eCases are described in further detail below. Student survey responses suggest that the integration of eCases in the PharmD curriculum is beneficial, with many respondents reporting that the eCases helped them learn and check their understanding of the material [9]. Although these perceptions are positive, it is unknown whether eCases truly enhance student comprehension as evidenced by improvements in academic performance. The benefits of instructional tools must be validated to demonstrate their value to students and instructors and justify ongoing development and implementation. The objective of this analysis was to identify the impact of eCase use on student performance in a cardiovascular therapeutics course. It was hypothesized that student use of the eCases would be associated with higher scores on course assessments.

## 2. Materials and Methods

### 2.1. General Design of eCases

eCases are unique instructional tools designed and developed at the University of Southern California Mann School of Pharmacy and Pharmaceutical Sciences (USC Mann). Modeled after “choose-your-own-adventure” (CYOA)-style cases, the eCases employ interactive, fictional patient cases that allow students to make therapeutic choices and observe the consequences of their decisions. The goal of the eCases is to improve the students’ ability to identify therapeutic problems and design optimal pharmacotherapy regimens, thereby targeting the higher domains of Bloom’s Taxonomy.

**Figure 1:**
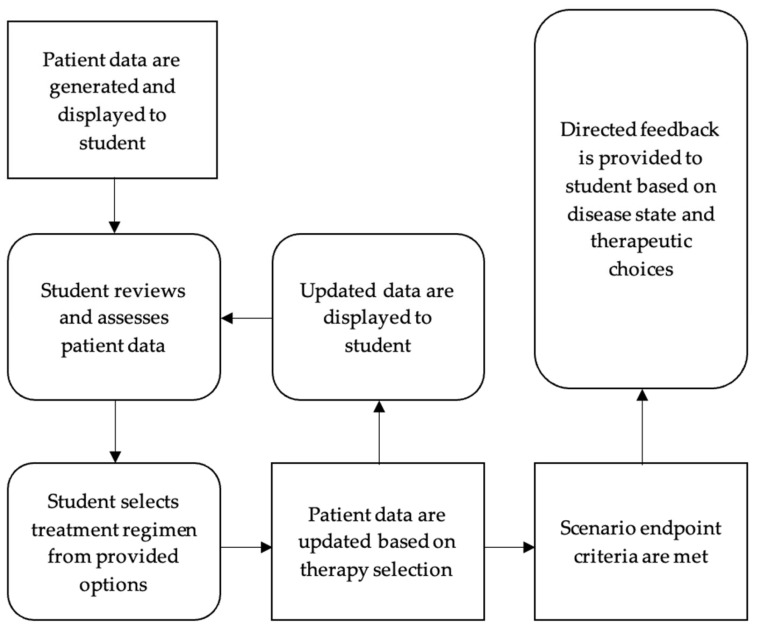
Basic diagrammatic representation of student progression through an eCase.

Each eCase focuses on a single disease state or topic, including various clinical presentations that may exist within that disease state. For example, the venous thromboembolism (VTE) eCase includes deep vein thrombosis (DVT) and pulmonary embolism (PE). In each eCase, students are presented with a randomly generated patient case based on a predetermined clinical presentation of the related disease state. Each patient has procedurally generated patient variables within a range of prespecified values. Examples of patient variables include age, comorbid conditions, vital signs, laboratory data, and diagnostic tests. After reviewing the patient’s data, the students are asked to initiate or adjust pharmacotherapeutic regimens. Choosing an appropriate regimen will either progress the case forward to another step or result in a “success”, while suboptimal regimens will result in a “failure”. Both “success” and “failure” scenarios stop the progression of the case and display specific feedback based on the student’s choices. Students are free to attempt a single patient case as often as desired to achieve success or explore how different therapeutic choices affect the patient. Students can also generate new patient cases to practice with different parameters within the same or different presentations of disease. The general flow of the eCases is depicted in Figure 1. Additional technical details regarding the design of eCases are described elsewhere [9].

The unique design of the eCases permits students to use the tool without faculty oversight, allowing for student-driven, independent learning. The randomization creates countless potential patient cases, leading to high levels of replay value.

### 2.2. Design and Implementation of eCases in the Cardiovascular Therapeutics Course

eCases for hypertension (HTN), VTE, and acute heart failure (AHF) were incorporated into the cardiovascular therapeutics course at our institution during the Fall 2020 semester. Students enrolled in the course were encouraged to use the eCases to augment their learning, but their use was optional and not linked to any grade incentive. Access to the eCases was provided to the students via the institution’s learning management system, permitting students to use the eCases at any time during the course. Integration of the eCases into class time was at the discretion of the affiliated lecturer. Students were provided instructions on how to access the eCase by each lecturer during their allotted class time. The lecturers for VTE and AHF used dedicated class time to demonstrate the related eCases.

Each disease state eCase was designed by the corresponding lecturer for student practice of the relevant pharmacotherapy and treatment guidelines, integrating factors such as comorbidities, renal/hepatic dysfunction, and drug–drug interactions. This design highlighted important concepts for student comprehension of the material and application to exam assessment items. The lecturers also determined criteria for successful progression through the eCase. To ensure relevance to student learning, lecturers aligned the criteria for success with lecture learning objectives and didactic content.

The HTN eCase highlights a patient with a new diagnosis Stage 1 or Stage 2 hypertension. Students are asked to identify the classification of hypertension and choose initial therapy from a list of antihypertensive drugs. Selection of an inappropriate therapy ends the eCase, while appropriate choices progress the case forward to a follow up visit. During that fictitious follow up visit, students may make further therapeutic adjustments, if desired, after which point, the eCase ends. Depending on the randomization, a wide range of therapeutic choices may result in successful progression through the eCase. Successful progression through this eCase occurs when the patient achieves a goal blood pressure with an appropriate treatment regimen.

The VTE eCase features a patient presenting to the hospital with a newly diagnosed DVT or PE, with or without the presence of shock. Students must identify the presentation of VTE and design a pharmacotherapeutic regimen from a list of options, including consideration of thrombolytics and anticoagulants. After selecting a regimen, students are brought to the end of the eCase. With many factors influencing appropriate treatment regimens, there are relatively fewer choices that result in successful progression through the eCase when compared to the HTN eCase. The criterion for success in this eCase is the selection of an appropriate pharmacotherapeutic regimen.

The AHF eCase allows students to practice the management of a patient with one of the following clinical presentations of AHF: warm and wet, cold and dry, or cold and wet. Based on the available data, students discern the presentation of AHF and select a treatment regimen targeted for specific hemodynamic endpoints. Students can choose to add and/or adjust medications from a list that includes diuretics (loop and thiazide), inotropes, vasoactive agents, and fluids, providing a multitude of therapeutic choices. After their recommendations are submitted, the patient data are updated and presented back to the students, allowing them to see the consequences of their decision-making in real time. This eCase progresses through multiple iterations of the same patient, with hemodynamic data changing based on the selected regimens. For example, a student may choose to increase the dose of a loop diuretic for a given patient and would receive updated hemodynamic information, upon which they can make further decisions. The eCase ends when predetermined criteria for success or failure are met or if the student takes too long to meet the success criteria. Of the three eCases described, the AHF eCase provides the highest level of randomization and narrowest window for success. Success in this eCase is achieved when target hemodynamic parameters are met. An example of the AHF eCase is provided in Appendix A.

The eCases were revised each year in response to student and faculty feedback, if necessary. Relevant changes included minor adjustments to the patient variables, medication choices, and feedback at the end of each eCase. No changes were made to the AHF eCase. The overall structure and content of the eCases remained consistent.

### 2.3. Study Design

This was a retrospective, observational cohort analysis of PharmD students at USC Mann enrolled in a cardiovascular therapeutics course from 2020 to 2022. This timeframe included transitions from in-class to a virtual format and then to flipped classroom learning as necessitated by the COVID-19 pandemic. The 2020 student cohort participated completely virtually, while the 2021 and 2022 cohorts completed a hybrid experience, with both in-person and virtual lectures. Aside from these transitions, the course teaching modalities remained consistent for all learners across all cohorts with minor adjustments for course content as needed for updates in treatment guidelines. Exams were cumulative and underwent annual revision such that each cohort received variations of similar exam items. Exams were administered in person except for in 2020, where students completed the exams virtually. Data on eCase usage characteristics and course performance were collected and deidentified after completion of the course then organized in aggregate. Students were grouped based on their use of eCases (eCase users vs. non-users). This research was deemed exempt from review by the Institutional Review Board.

The following data were collected about eCase use: user ID (nonidentifiable), disease state, disease presentation, number of unique patient cases generated, number of attempts, and whether each attempt was successful. Each time a student started an eCase, a unique patient case was generated. An attempt was defined as a student’s complete progression through the patient case and was considered successful if the student met the prespecified criteria for that eCase.

The student performance metrics included the overall course score and scores on the midterm and final exams. Performance data were also collected for content-specific exam items related to the topics of HTN, VTE, and AHF. These metrics were aggregated and analyzed based on the use or non-use of the eCases and separated by year (2020, 2021, 2022). This process was repeated for student performance on content-specific exam items (e.g., performance on VTE exam items by use of the VTE eCase). Additional analyses were completed to assess the rate of eCase use across the quartiles of performance metrics. The Mann–Whitney U test was used for continuous variables and chi-square tests were used for categorical variables. *p*-values less than 0.05 were considered significant for the Mann–Whitney U test, and those less than 0.0083 were considered significant for the chi-square tests to account for multiple comparisons. All statistical analyses were performed using SPSS Statistics v.29.0 (IBM, Armonk, NY, USA).

## 3. Results

### 3.1. eCase Use Characteristics

A total of 562 students were included, with 199, 193, and 170 students in the 2020, 2021, and 2022 cohorts, respectively. The characteristics of eCase usage are shown in Table 1. Of the 562 students, 322 (57.3%) used at least one of the available eCases. Among the eCase users, 129 (40.1%) used only one eCase, 122 (37.9%) used two eCases, and 71 (22.0%) used all three eCases. Notably, no students in the 2022 cohort used the HTN eCase. The median numbers of both the unique patient cases attempted and attempts per student were higher in the 2021 and 2022 cohorts than in the 2020 cohort.

### 3.2. Exam and Overall Course Performance

Table 2 shows the performance of eCase users compared with eCase non-users on the midterm exam, final exam, and in the course overall. There was no difference in course or exam performance between the eCase users and non-users in the 2020 and 2021 cohorts. In the 2022 cohort, students who used an eCase had significantly higher overall course scores compared with non-users (83.6% vs. 79.7%, *p* = 0.002), likely driven by their higher scores on the final exam (75.0% vs. 67.7%, *p* < 0.001). In a sensitivity analysis that considered eCase users who attempted all three eCases, the results remained the same. 

In the 2020 and 2021 cohorts, there were no differences in the rates of eCase use between students in the top and bottom quartiles of the course, midterm exam, and final exam scores. In the 2022 cohort, more students who scored in the top quartile on the final exam used an eCase compared with students who scored in the bottom quartile (35/48 [72.9%] vs. 14/40 [35.5%], *p* < 0.001). eCase users in the 2022 cohort were more likely to be top performers than bottom performers on the final exam (OR 5.0, 95% CI: 2.0 to 12.4). The rates of eCase use in the 2022 cohort were not different between the top and bottom quartiles of performers in the course or on the midterm exam.

### 3.3. Hypertension Exam Item Performance

Table 3 shows the students’ performance on content-specific exam items based on their use of the related disease state eCase. There was no difference in performance on HTN-specific exam items between HTN eCase users and non-users in any year. Similarly, there was no difference in the rate of HTN eCase use between students in the top and bottom quartiles of HTN exam item scores in any cohort.

### 3.4. Venous Thromboembolism Exam Item Performance

For VTE-specific exam items, VTE eCase users received significantly higher scores compared with non-users in the 2021 cohort, but not in the 2020 or 2022 cohorts. In the 2021 cohort, there were more VTE eCase users among the students who received scores in the top quartile compared with the bottom quartile (21/51 [41.2%] vs. 8/41 [19.5%], *p* = 0.026). The rates of VTE eCase use were similar among students in the top and bottom quartiles in the 2020 and 2022 cohorts.

### 3.5. Acute Heart Failure Exam Item Performance

Students in the 2021 and 2022 cohorts who used the AHF eCase had higher scores on the AHF exam items compared with non-users. This difference was not found in the 2020 cohort. In the 2021 cohort, the rates of AHF eCase use were significantly higher among students who scored in the top quartile compared with the bottom quartile on the AHF exam items (28/48 [58.3%] vs. 15/51 [29.4%], *p* = 0.004). In the 2022 cohort, there were more AHF eCase users in the top quartile compared with the bottom quartile of scores for the AHF exam items (31/48 [64.6%] vs. 18/56 [32.1%], *p* = 0.004). When all three cohorts were combined, students who used the AHF eCase had higher odds of being in the top quartile than the bottom quartile of scores on AHF exam items (OR 2.1, 95% CI: 1.3 to 3.3).

## 4. Discussion

Novel and innovative instructional tools allow students to engage with material in different ways but should demonstrate value through measurable improvements in learning [10]. This research evaluated the impact of eCase use on student performance in a PharmD cardiovascular therapeutics course, finding that the use of eCases may improve student performance as measured by assessment scores. However, the determination of the best approach, format, or subject matter that adds the most value requires further study.

Prior studies on the integration of CYOA-style cases into pharmacy education have shown positive student perceptions of the teaching tool and improvements in knowledge [11,12,13,14,15,16]. In their study, Aguiniga et al. describe the impact of CYOA-style cases on assessment scores [11]. The authors incorporated CYOA activities for chronic obstructive pulmonary disease (COPD) and VTE in dedicated class time. The CYOA activity was immediately preceded by a pre-quiz and followed by a post-quiz. Knowledge in the content area was also tested on an exam later in the course. The authors reported a decrease in VTE assessment scores and an increase in COPD assessment scores after completion of the CYOA activity. Note that all students in the study participated in the CYOA activity and no comparisons were made between students who participated and those who did not.

Vadiei and Lee described the integration of a CYOA-style case on depression and anxiety into a psychiatric pharmacotherapy course [12]. The CYOA activity included five predefined treatment choices. A pre-activity and post-activity knowledge assessment was administered to the participating students. The authors reported a higher percentage of correct responses in the post-activity assessment as compared to the pre-activity assessment. Like the study by Aguiniga et al., data were only available for students who participated in the CYOA activity.

This current analysis is unique in providing a comparison between students who used the instructional tool compared to those who did not. Using exam and course scores as performance metrics, there appeared to be an association between higher final exam scores and eCase use in certain cohorts that drove higher overall course grades. However, this effect was not consistent across all cohorts studied.

The difference in eCase efficacy across cohorts is likely multifactorial. The 2020 cohort completed exams virtually, which may have influenced the integrity of the test-taking environment. As such, any impact of the eCases may have been masked in that cohort. The differences seen may also reflect revisions to the eCases over time, including changes to the feedback provided to the students at the end of each eCase, delivering more specific comments regarding therapeutic choices made by the student. Faculty may also have gained greater familiarity and practice integrating the eCases into lesson plans over time, particularly for the VTE and AHF eCases. As such, their utility as instructional tools likely improved over time, leading to the results seen in the 2022 cohort.

There were notable differences in student use of disease state eCases and performance on the related disease state exam items. Although the use of the HTN and VTE eCases did not appear to influence performance on the HTN and VTE exam items, the AHF eCase showed a more robust effect on AHF exam item scores. Notably, as AHF questions appeared only on the final exam, the improved performance on these questions may have contributed to the higher final exam scores among eCase users. The higher scores seen on AHF questions likely reflect the design variations between the different eCases. As noted above, the AHF eCase has a high degree of randomization in therapeutic regimens, creating multiple opportunities for students to engage with the material. The inclusion of several steps of progression through the AHF eCase creates additional chances for student engagement. Furthermore, students can see the impact of discrete therapeutic choices on a patient. These are all factors that likely contributed to the benefit seen with use of the AHF eCase and should shape future development of eCases or other interactive patient cases.

A key goal in designing and developing the eCases was creating opportunities for PharmD students to practice their problem-solving, clinical reasoning, and clinical decision-making skills. Despite the widely recognized importance of these skills, many educators struggle with successfully teaching them in the didactic setting [1]. If a well-designed eCase can provide an avenue for students to nurture these skills, they may serve as an effective instructional modality across the PharmD curriculum. Such a potential tie warrants further investigation.

## 5. Limitations and Future Work

There are several limitations to this research. The successful integration of eCases into a PharmD curriculum requires an investment of time and resources that may not be possible in all programs. However, the required investment for developing eCases may be much lower than comparable instructional tools [13]. In a review by Wettergreen et al., faculty time commitment to developing CYOA-style cases exceeded 50 h in most cases. In contrast, the development of the eCase required less than four hours of faculty time [9]. Second, the overall course score was used as a performance marker. There were low-stakes assessments and assignments outside of the exams that contributed to the course grade, which could have influenced this marker. As these other assessments and assignments could be completed with access to resources, it is unlikely that there were substantial differences in scores between eCase users and non-users, and thus it is doubtful that this affected the results of this analysis.

Importantly, this analysis encompassed three separate cohorts and included transitions from in-class learning to virtual learning, and finally, to a flipped classroom model. Additionally, these cohorts received different versions of the assessments. Though comparisons between cohorts or combining cohorts may be of interest, these comparisons would not be accurate due to these differences.

In the 2022 cohort, no student used the HTN eCase, and it is unknown why there was a lack of engagement with this specific eCase in that cohort. This finding confounds the interpretation of the results and limits any statement that may be made about the impact of that specific eCase. Finally, as eCase use was neither mandatory nor incentivized, eCase use was student-driven. The students who used the eCases may have sought out additional supplemental resources because they were more engaged in the course, which may have contributed to the results of this analysis.

Despite these limitations, this research has notable strengths. This research included students across three years, providing a large data set for evaluation. In contrast, previous studies were limited to evaluation of a single class year [13]. Additionally, prior studies that have measured student performance included only students who participated in the activity and primarily evaluated differences in pre-activity and post-activity assessments [13]. This current analysis included both students who used and did not use the eCase, allowing for comparisons of student performance based on eCase use. Such a comparison may better reflect the true impact of an instructional tool.

This research highlights the potential of eCase as an instructional tool that may improve student performance in a cardiovascular therapeutics course. As the effect was inconsistent across different student cohorts and disease states, thoughtful eCase design will be important to maximize their effectiveness. Additional investigations should elucidate which eCase characteristics are most vital for their effectiveness to inform the development and integration of eCases in other areas.

## 6. Conclusions

Student use of eCases was associated with improved performance metrics in a cardiovascular therapeutics course, but the effect was variable across cohorts and eCase topics. This effect was most notable for the AHF eCase and performance on AHF exam items. The results of this analysis support the continued development and integration of eCases into the PharmD curriculum while highlighting the importance of eCase design for its educational utility. Further research is warranted to identify the specific characteristics of eCases that lead to their beneficial effects.

## Figures and Tables

**Table 1:** Number and percentage of students using eCases with attempt characteristics by year ^†^.

	2020	2021	2022
Student eCase use, n (%)			
Overall	118 (59.2)	110 (57.0)	94 (55.2)
HTN eCase	88 (46.3)	67 (34.7)	0 (0.0)
VTE eCase	77 (38.7)	61 (31.6)	72 (42.4)
AHF eCase	60 (30.2)	88 (45.6)	73 (42.9)
Number of students who used zero to all three disease state eCases, n (%)			
No eCases	81 (40.7)	83 (43)	76 (44.7)
1 eCase	44 (22.1)	42 (21.8)	43 (25.3)
2 eCases	41 (20.6)	30 (15.5)	51 (30.0)
3 eCases	33 (16.6)	38 (19.7)	0 (0.0)
Unique eCase patients attempted per student, median (range) ^‡^	3 (1–47)	6 (1–117)	5 (1–66)
Number of attempts per student, median (range) ^‡^	4 (1–79)	16 (1–170)	16 (1–193)

AHF: acute heart failure; HTN: hypertension; VTE: venous thromboembolism. ^†^ Number of students: 2020 (N = 199), 2021 (N = 193), 2022 (N = 170). ^‡^ Includes students who used any disease state eCase.

**Table 2:** Student course and exam performance based on eCase use ^†^.

	eCase Users	eCase Non-Users	*p*-Value
2020			
Course score, %, mean (SD)	91.2 (5.5)	91.6 (5.5)	0.564
Midterm exam score, %, mean (SD)	92.9 (6.8)	93.8 (6.5)	0.318
Final exam score, %, mean (SD)	71.1 (8.0)	71.3 (8.1)	0.832
2021			
Course score, %, mean (SD)	85.8 (6.9)	85.6 (6.5)	0.880
Midterm exam score, %, mean (SD)	78.0 (10.5)	78.5 (10.1)	0.695
Final exam score, %, mean (SD)	77.8 (9.7)	75.4 (10.4)	0.102
2022			
Course score, %, mean (SD)	83.6 (8.1)	79.7 (9.0)	0.002
Midterm exam score, %, mean (SD)	75.9 (12.6)	73.8 (13.1)	0.296
Final exam score, %, mean (SD)	75.0 (11.9)	67.7 (12.5)	<0.001

^†^ Any student who used any of the eCases was considered an eCase User.

**Table 3:** Performance on content-specific exam questions based on disease state-specific eCase use.

Content-Specific Exam Items	Disease State-Specific eCase User	Disease State-Specific eCase Non-User	*p*-Value
HTN exam items correct, %, mean (SD)			
2020 cohort	70.3 (7.9)	70.7 (7.3)	0.618
2021 cohort	60.8 (12.6)	64.1 (11.3)	0.094
2022 cohort ^†^	0	61.2 (13.3)	-
VTE exam items, %, mean (SD)			
2020 cohort	80.9 (14.3)	82.1 (13.3)	0.583
2021 cohort	73.9 (13.4)	68.2 (13.3)	0.005
2022 cohort	67.4 (14.4)	66.2 (17.3)	0.595
AHF exam items, %, mean (SD)			
2020 cohort	57.2 (12.6)	55.8 (13.5)	0.695
2021 cohort	67.1 (14.5)	61.7 (14.5)	0.013
2022 cohort	65.4 (19.9)	54.0 (19.8)	<0.001

AHF: acute heart failure; HTN: hypertension; VTE: venous thromboembolism. ^†^ No students in the 2022 cohort used the HTN eCase.

**Figure A1:**
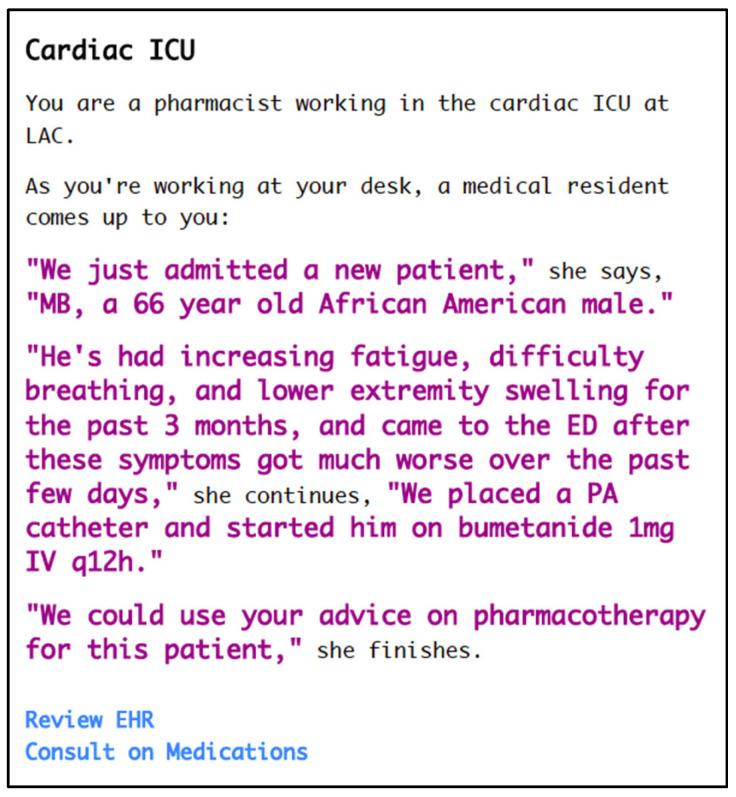
Depiction of the initial patient scenario provided to the student.

**Figure A2:**
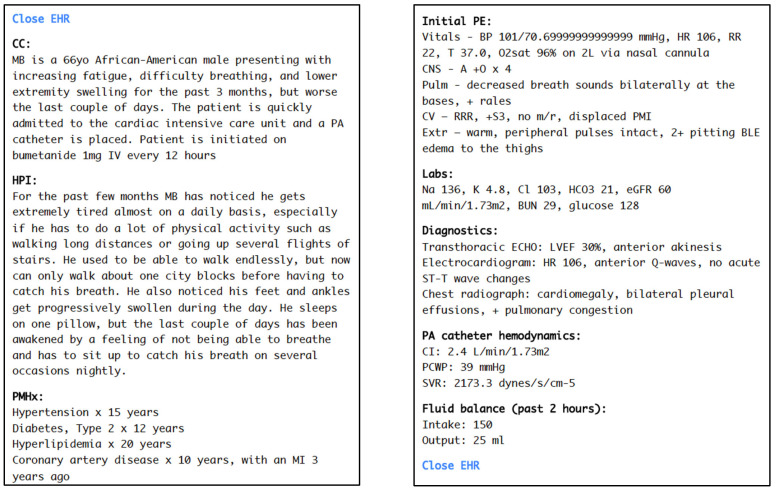
Depiction of the example patient’s profile.

**Figure A3:**
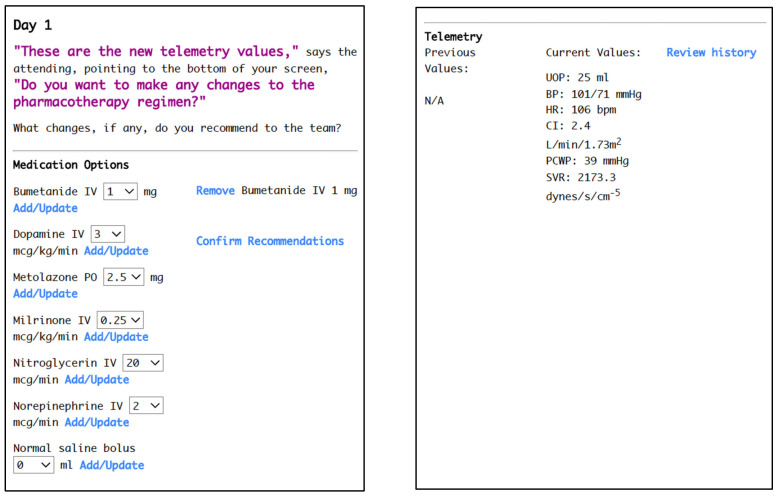
Depiction of the initial decision point for adjusting the patient’s therapeutic regimen.

**Figure A4:**
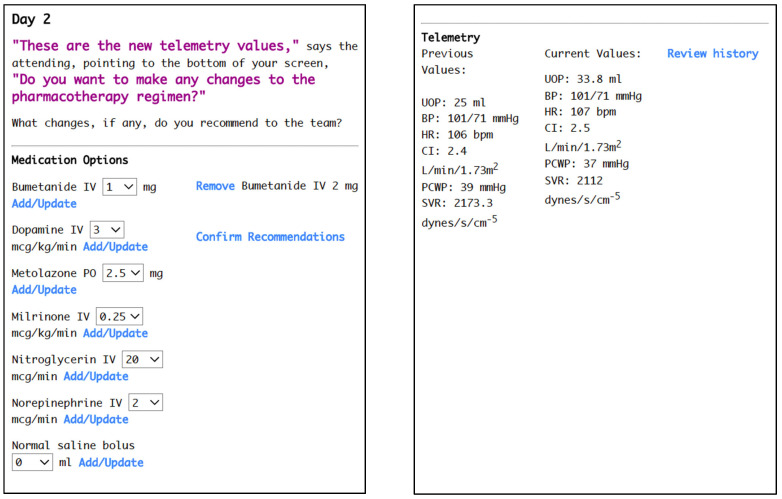
Depiction of a subsequent decision point for adjusting the patient’s therapeutic regimen.

**Figure A5:**
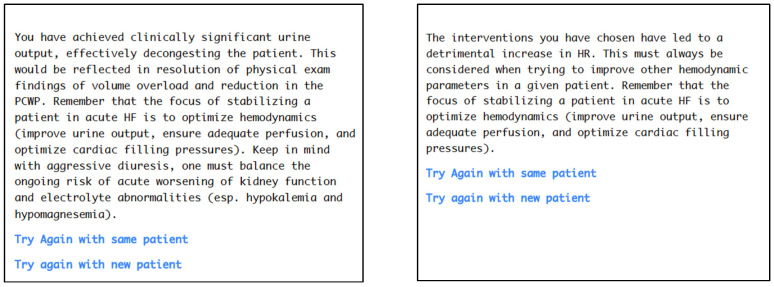
Depiction of feedback provided to the student after successful and unsuccessful completion of the eCase.

## Data Availability

The data presented in this study are not readily available because the data are part of an ongoing study. Requests to access the data should be directed to the corresponding author.

## Abbreviations

The following abbreviations are used in this manuscript:

CYOA	Choose-your-own-adventure
HTN	Hypertension
VTE	Venous thromboembolism
AHF	Acute heart failure

## References

[1] Persky A.M., Medina M.S., Castleberry A.N. (2019). Developing Critical Thinking Skills in Pharmacy Students. Am. J. Pharm. Educ..

[2] Richards J.B., Hayes M.M., Schwartzstein R.M. (2020). Teaching Clinical Reasoning and Critical Thinking: From Cognitive Theory to Practical Application. Chest.

[3] Schmidt H.G., Mamede S. (2015). How to improve the teaching of clinical reasoning: A narrative review and a proposal. Med. Educ..

[4] Persky A.M., McLaughlin J.E. (2017). The Flipped Classroom—From Theory to Practice in Health Professional Education. Am. J. Pharm. Educ..

[5] Gleason B.L., Peeters M.J., Resman-Targoff B.H., Karr S., McBane S., Kelley K., Thomas T., Denetclaw T.H. (2011). An active-learning strategies primer for achieving ability-based educational outcomes. Am. J. Pharm. Educ..

[6] Torralba K.D., Doo L. (2020). Active Learning Strategies to Improve Progression from Knowledge to Action. Rheum. Dis. Clin. N. Am..

[7] Vyas D., Ottis E.J., Caligiuri F.J. (2011). Teaching clinical reasoning and problem-solving skills using human patient simulation. Am. J. Pharm. Educ..

[8] Rotellar C., Cain J. (2016). Research, Perspectives, and Recommendations on Implementing the Flipped Classroom. Am. J. Pharm. Educ..

[9] Morningstar-Kywi N., Kim R.E. (2021). Using Interactive Fiction to Teach Clinical Decision-Making in a PharmD Curriculum. Med. Sci. Educ..

[10] Persky A.M., Lee E., Schlesselman L.S. (2020). Perception of Learning Versus Performance as Outcome Measures of Educational Research. Am. J. Pharm. Educ..

[11] Aguiniga A.M., Phillips H., Howard M.L. (2024). Effect of Choose-Your-Own-Adventure (CYOA) Activities on Pharmacy Student Knowledge. Am. J. Pharm. Educ..

[12] Vadiei N., Lee J.K. (2022). An innovative approach to teaching depression and anxiety medication management: Virtual choose your own adventure, psychiatry edition. Ment. Health Clin..

[13] Wettergreen S.A., Scott C., Auten M., Kiles T.M., Litten K., Scott D., Stewart M.P. (2024). A meta-narrative review of choose-your-own-adventure style patient case activities in pharmacy education. Curr. Pharm. Teach. Learn..

[14] Kiles T.M., Hall E.A., Scott D., Cernasev A. (2021). Enhancing Student Knowledge of Diabetes through Virtual Choose Your Own Adventure Patient Case Format. Pharmacy.

[15] Scott D., Cernasev A., Kiles T.M. (2021). Reimagining Pharmacy Education through the Lens of a Choose Your Own Adventure Activity-A Qualitative Evaluation. Pharmacy.

[16] Osae S.P., Palmer R., Smith K., Misher A. (2022). Lessons learned from a formative study evaluating student pharmacists’ experience with a case-based learning “choose your own adventure” activity. Curr. Pharm. Teach. Learn..

